# Morphological effect of side chain on H_3_O^+^ transfer inside polymer electrolyte membranes across polymeric chain via molecular dynamics simulation

**DOI:** 10.1038/s41598-020-77971-6

**Published:** 2020-12-16

**Authors:** JinHyeok Cha

**Affiliations:** Institute of Fundamentals and Advanced Technology, Hyundai Motor Company, 37 Cheoldobangmulgwan-ro, Uiwang-si, Gyeonggi-do 16082 Republic of Korea

**Keywords:** Nanoscale materials, Nanoparticles, Structural properties

## Abstract

Performance and durability of polymer electrolyte membrane are critical to fuel cell quality. As fuel cell vehicles become increasingly popular, membrane fundamentals must be understood in detail. Here, this study used molecular dynamic simulations to explore the morphological effects of perfluorosulfonic acid (PFSA)-based membranes on ionic conductivity. In particular, I developed an intuitive quantitative approach focusing principally on hydronium adsorbing to, and desorbing from, negatively charged sulfonate groups, while conventional ionic conductivity calculations featured the use of mean square displacements that included natural atomic vibrations. The results revealed that shorter side-chains caused more hydroniums to enter the conductive state, associated with higher ion conductivity. In addition, the hydronium path tracking showed that shorter side-chains allowed hydroniums to move among host groups, facilitating chain adsorption, in agreement with a mechanism suggested in earlier studies.

## Introduction

The hydrogen fuel cell was introduced by William Robert Grove in 1839^1^. After 180 years, potential applications continue to engage many researchers in various industrial fields^2^. In particular, the Japanese Hydrogen Society, established in 2010, has created great interest in hydrogen-based energy. Over 50 global industries have joined the Hydrogen Council, which was established in 2017 by an initial 13 industries in Davos, Switzerland. The Hyundai Motor Company, Toyota, and Honda today produce four types of hydrogen fuel cell vehicles. Basically, a hydrogen fuel cell generates electrical power via three simple steps: (i) dissociation of hydrogen to the proton and electron; (ii) conduction of electrons and protons through electronic channels and an electrolytic membrane, respectively; and, (iii) creation of water when hydronium (H_3_O^+^) and an electron meet/oxygen. The several types of hydrogen fuel cell include the polymer electrolyte membrane fuel cell (PEMFC), the alkaline cell, the phosphoric acid fuel cell, the molten carbonate cell, the direct methanol fuel cell, and the solid oxide cell^3^. PEMFCs are commonly used in vehicles because they operate better at relatively low temperatures than do other fuel cells^4^. PEMFCs feature a stack and a balance of plant (BOP); the stack (serially connected unit cells) plays the role of an internal combustion system that generates electricity from hydrogen and oxygen. Each unit cell features a bipolar gas diffusion layer and a membrane electrode assembly (MEA) directly responsible for the electrochemical reaction. All electrochemical reactions of the MEA (hydrogen dissociation at the anode, proton transport through a polymer electrolytic membrane, and water production at the cathode) are nanoscale phenomena. Many studies have explored these steps via first-principles or molecular dynamic (MD) simulations^5–8^.

Various types of energy loss occur inside the MEA during electrochemical reactions^9^. The transport of dissociated protons inside polymer electrolyte membranes critically influences fuel cell efficiency. Such membranes may be fabricated from hydrocarbon-based polymers (polyphenylene oxide, polyarylene ether ketone, or polybenzimidazole), or from perfluorinated polymers with functionalized groups such as sulfonic acid. The latter membrane types are preferred given their excellent chemical, thermal, and mechanical properties^10^. Nafion is the electrolyte membrane most commonly used in present-day fuel cells; this is a perfluorosulfonic acid (PFSA)-based membrane developed by DuPont Inc. more than 40 years ago^11^. The morphology of Nafion has been phenomenologically studied under a variety of operating conditions. In particular, there have been simulation efforts to explore the effects of on structural and dynamical properties by the morphology^12,13^. Of that, modification of the acidic group attached to the membrane, or changing of the equivalent weight (EW) by shortening the side-chains or tuning the backbone, have an impact on membrane durability and conductivity^14–16^. Paddison et al. used a first-principles approach to show that backbone length and conformation, flexibility, and side-chain structures played important roles in proton dissociation and transport^17–22^. In particular, the inclusion of terminal sulfonate groups engaging in side-chain bonding critically affects proton transfer inside the membrane^23^. Dow Chemical synthesized PFSA-based membranes with short side-chains; conductivity improved compared to that of Nafion membranes^24–26^.

In contrast, a recent experimental study found that short side-chains did not significantly affect water diffusion, proton transport, or hydrophilic/hydrophobic separation^27^. An all-atom MD simulation found that short side-chains were associated with greater proportions of free H_3_O^+^ ions, affording higher ionic conductivity^28^. Brandell et al. concluded that Nafion exhibited higher ionic conductivity than membranes with shorter or longer side-chains^29^. The effects of membrane morphology on ionic conductivity have been intensively researched over more than four decades. Nonetheless, structural influences on H_3_O^+^ conductance inside PEMFCs remain poorly understood. An unconventional approach incorporating more fundamental details is required.

In this study, I used MD simulations to investigate the morphological effects of pendant-like side-chains bearing terminal sulfonate groups on ionic conductivity. I employed a novel quantitative evaluation method focusing principally on H_3_O^+^ adsorption/desorption to/from negatively charged sulfonate groups, while conventional calculation of ion conductivity derived from the mean square displacement (MSD) method considering all natural atomic vibration. I found that a shorter side-chain rendered more hydroniums conductive, thus enhancing ionic conductivity. By tracking the hydronium paths, I concluded that a shorter side-chain allows protons to jump among chain host groups, facilitating adsorption.

## Simulation methodology

### Models of polymer electrolyte membranes used in fuel cells

Proton transport is caused by two representative mechanisms, Grotthuss mechanisms and vehicular mechanisms^30^. The Grotthuss mechanism can be studied by ab initio molecular dynamics simulations which provide the most realistic description, but it requires the highest computational costs. To improve the efficiency of the computation, semi empirical methods^31,32^ and hybrid quantum/classical protocols have been proposed^33^. A multistate empirical (MS-EVB)^34–36^ and reactive force field^37,38^ approaches are capable of explicit proton transfer. During the performance of classical molecular simulation, the proton transfer is evaluated in an effective potential energy landscape extracted from ab initio calculations of model transfer events. Although the size and timescale of the system are larger than in ab ignition calculations, an order of magnitude is smaller than in classical molecular dynamics simulations^39^. In addition, Q-HOP method^40^ with required careful parameterization from accurate quantum chemical calculations for hopping events reproduces the kinetic picture of proton transfer well that excess proton is covalently bound to a specific atom, which, however, is not microscopically reversible and consequentially cannot produce correct thermodynamic ensembles^39^. In this study, I employed nonreactive force field method to describe proton exchange phenomenon in larger length and time scale. Although the result hardly includes the effect of Grotthuss mechanism on conductivity, the condition of composed system with relatively dried state in this study regards vehicular mechanism to be much more influential on ionic conductance considering PFSA nanostructural models^41,42^.

**Figure 1:**
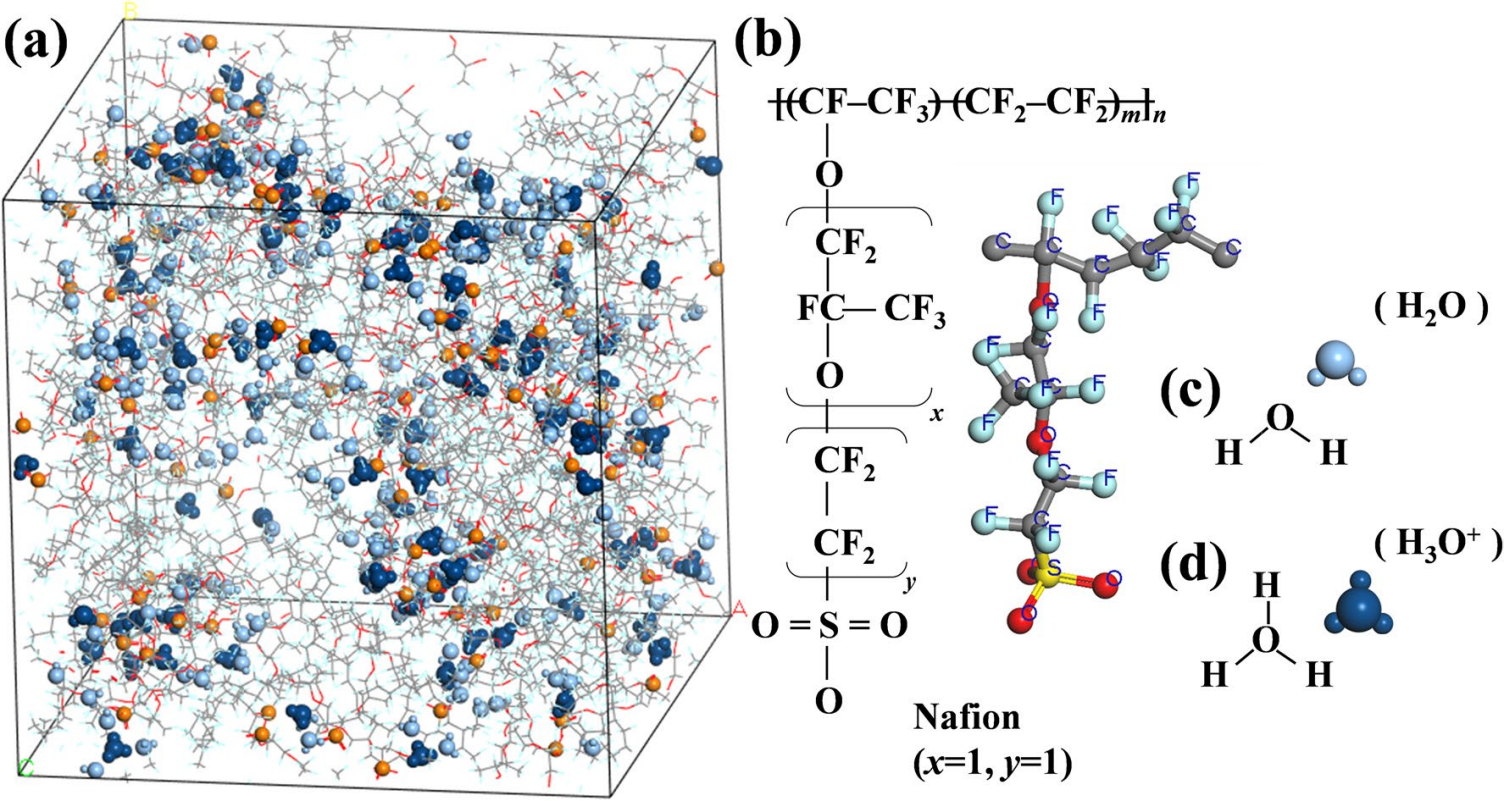
(**a**) A snapshot of molecular dynamics simulation of a polymer electrolyte membrane fuel cell (PEMFC) in a hydrated state (*λ* = 3); (**b**) perfluorinated sulfonic-acid (Nafion); (**c**) H_2_O; and, (**d**) protonated hydronium (H_3_O^+^).

When engaging in MD simulations of polymer-based systems, the use of at least 10 chains each containing over 10 monomers is conventionally regarded as representative^43^. In this study, all MD simulations contained 10 polymeric chains, each with 10 repeat monomers at hydration level (*λ*) 3; these transported H_3_O^+^ (Fig. 1a). The chemical structure of a PFSA-based ionomer can be divided into a backbone (–CF_2_–) and pendant–OCF_2_CF(CF_3_)OCF_2_CF_2_SO_3_– groups that become shorter or longer as *x* and *y* vary (Fig. 1b). The sulfonate group (–SO_3_–) on the end of the side-chain (i.e., the “pendant” on the chain) is negatively charged and attracts water and H_3_O^+^ molecules. In general, a modifying index is used to create side-chains of a desired structural length; it is possible to change both the *x* and *y* indices. The end of each chain is terminated by fluorine. In addition, I assumed that all sulfonated groups were ionized. The EW of the Nafion employed in this study was 1147. The *λ* is important; here, it is presented as the ratio of H_2_O (Fig. 1c) including H_3_O^+^ (Fig. 1d) molecules to sulfonated groups. When transporting H_3_O^+^, the membrane must be hydrated; it then becomes a transportation channel. The *λ* of the fuel cell PEM critically affects ionic conductivity, endurance, and even the mechanism of H_3_O^+^ transportation.

### Simulation methods

The factor primarily affecting ionic conduction is the structural length of the PFSA side-chain. Although the length ranges from 4.66 × 4.66 × 4.66 to 5.98 × 5.98 × 5.98 Å^3^, the effects of length size differences are negligible in polymeric systems if density is identical across all simulations. The number of atoms in a single chain with 10 PFSA monomers was 582, 682, 982, and 1282, corresponding to *y* values of 0, 1, 4, and 7, respectively. A single PFSA chain contains 10 side-chains, i.e., 10 negatively charged sulfonate groups; I added 100 positively charged H_3_O^+^ ions to each system to hold the equivalent net charge constant. Thus, each system featured 200 H_2_O molecules and thus a *λ* of 3, as described in “Models of polymer electrolyte membranes used in fuel cells” section.

All MD simulations were based on the DREIDING force field, which usefully predicts the structures and dynamics of various organic, biological, and inorganic molecules engaging in simple hybridization^44^. The force field features single force constants for each bond, angle, and inversion; and six different torsional barriers. All the parameters and charges assigned on each atoms are listed in Supplementary Information^43^ (Supplementary Tables S1, S2, Fig. S1). To build high density up to 1.7 g/cm^3^ PEMFC for MD simulations, I employed the annealing to reach targeting density. The annealing process that the simulation system begun with the density of 0.6 g/cm^3^ became to be the desired density by *NPT* ensemble to minimize energy of the system effectively (Supplementary Fig. S2), where *N*, *P*, and *T* are the number of atoms, pressure, and temperature of the system, respectively. Each polymer chain spread uniformly at the beginning interacts each other during the annealing process. Gradually, it formed clustering with gyration by attractive interaction between ionic domain of the polymer. Eventually, it reached to the targeting density with hydration.

Annealing was simulated from 300 to 550 K by increasing or lowering the temperature at a ramp of 50 ps per *NPT* ensemble cycle. Nosé-Hoover-Langevin algorithm^45^ was used for thermostat to control the temperature of the simulations which contains Langevin friction and noise term to the thermostat variable for the efficiency of simulation time. (Supplementary Method S1) To control pressure of the systems, Beredsen method^46^ was employed as a barostat for *NPT* simulation that changes the coordinates of the particles and the size of the unit cell by employing re-scaling factor. (Supplementary Method S2) The cycle was repeated until the simulated density attained the desired 1.7 g/cm^3^; the initial setup featured 20 cycles. Simulation was initiated using the modeling process described above for 500 ps at 300 K; I employed *NVT* ensemble to identify ionic molecular behavior. Although the annealing process led to energy stabilization of the system, I excluded data from the initial 200 ps by considering unexpected molecular behavior by different condition such as exchanged ensembles.

DFT calculations were implemented using the Dmol^3^ program. The exchange–correlation functional was Perdew-Burke-Emzerhog (PBE) functional with the generalized gradient approximation (GGA)^47^, and the Becke’s 3 parameter functionals and Lee, Yang and Parr’s correlation functionals (B3LYP)^48,49^. The spin-polarized calculations were performed using double numerical basis set with polarization functions (DNP), and triple basis set with polarization functions (TNP). All electron relativistic effects were included for the treatment of core electrons in the models.

All simulation models were three-dimensional periodic structures constructed using “Materials Studio 2016” software (BIOVIA Software Inc., San Diego, CA, USA).

## Results and discussion

### Numerical evaluation of ion conductivity

Ion conductivity critically affects PEMFC performance. To evaluate molecular conductivity during vehicular conduction by a simulated polymer, it is necessary to define a diffusion coefficient, the so-called diffusivity (*D*), which is given by:

1 $$ D = \frac{{1}}{{6}}\mathop {lim}\limits_{{\Delta t \to \infty }} \frac{dMSD}{{d\Delta \text{t}}} $$

**Figure 2:**
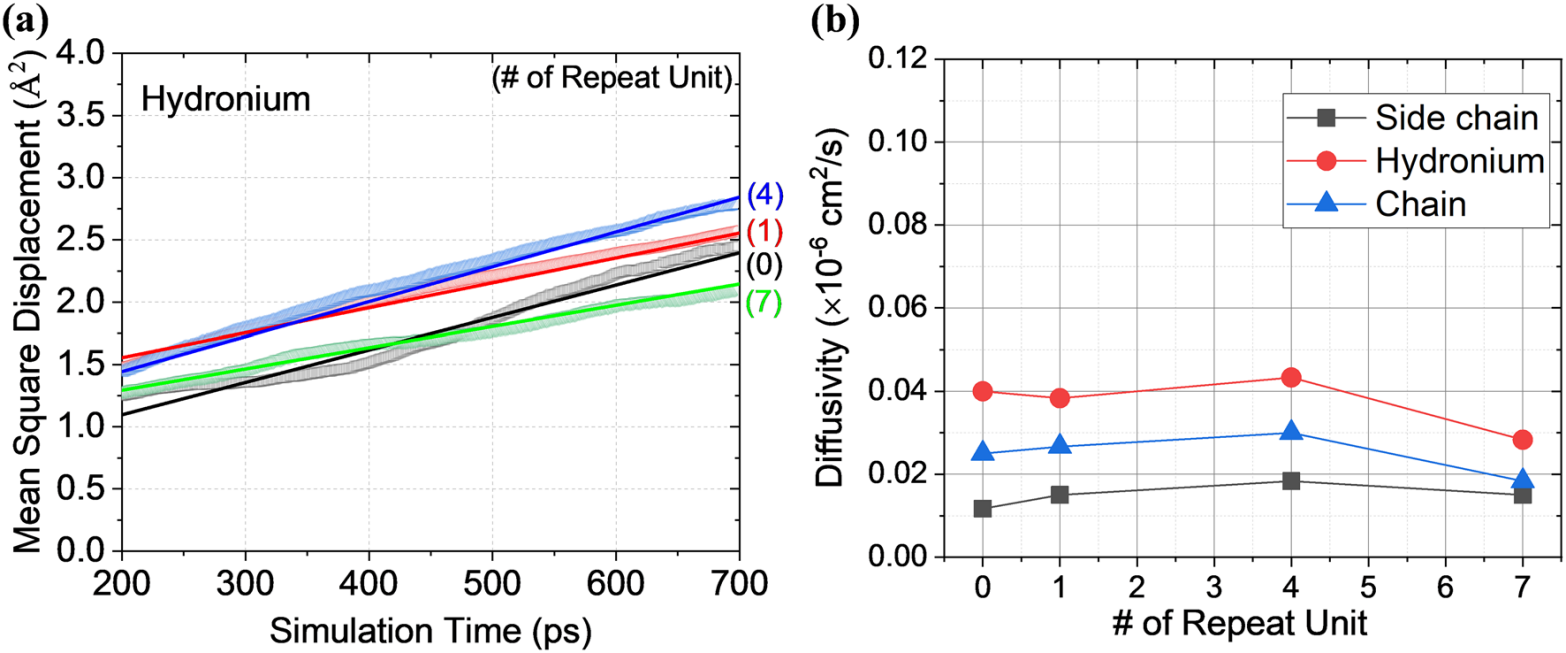
(**a**) Mean square displacement (MSD) of hydronium (H_3_O^+^), and derived (**b**) diffusion coefficient of all the molecules.

MSD is given by:

2 $$ MSD \equiv \left\langle {{(}x - x_{{0}} {)}^{{2}} } \right\rangle = \frac{1}{T}\sum\limits_{t = 1}^{T} {\left( {x{(}t{)} - x_{0} } \right)^{2} } $$

where *T* is the average time and *x*_0_ is the reference position of the particle. The H_3_O^+^ ion is attracted to the negative sulfonate group at the end of a side-chain, and then moves to another sulfonate group. As I do not consider the ionic conductance by Grotthuss mechanism, the diffusion coefficient plays a critical role in terms of ion conductivity in MD simulations; as defined by:

3 $$ Ion \,conductivity = \frac{{e^{2} }}{{Vk_{b} T}}D $$

where *e*, *V*, *K*_*b*_, *T*, and *D* are the elementary charge, volume, Boltzmann constant, temperature, and diffusion coefficient of the system, respectively^50^. Figure 2a shows the MSD of hydronium with the length of side chain. More specifically, I also investigated the molecular diffusion coefficient derived from MSD to find kinetic behavior of each molecules (Fig. 2b, Supplementary Fig. S3). The results reveal that the diffusivity of all the element at various length of side chains seems no dependency on the number of repeat unit. Liu et al.^51^ reported the morphological effect of side chain on diffusion coefficient at various hydrated membrane that diffusion coefficient derived from mean square displacement (MSD) decreased with increase of length of side chain. However, MSD-based analysis is limited to distinguish diffusion coefficient with respect to the length of side chain at low hydrated number (*λ* = 3). For instance, 678, 778, 878, and 978 of EW corresponded to diffusion coefficient of 0.05, 0.06, 0.05, and 0.05 cm^2^/s, respectively, with few differences defined. To understand structural effect of the side chain on molecular behavior, it needs more institute evaluation method with different approach from conventional perspective to clarify the mechanism for hydronium conduction such as inter- or inter conduction in the polymeric system.

In addition, the interactions between atoms of non-covalently bound molecules during MD simulations are associated with natural vibrations that create the most stable van der Waals distance. Thus, it is possible that conventional analyses of ionic conductivity using relationships derived from the diffusion coefficient and the MSD (as employed above) include all distances imparted by these vibrations. Ultimately, longer simulation times are required to distinguish natural vibrations from real ionic movements, or the data obtained from larger system of the simulations is needed for the desired results.

Here, I performed a numeric evaluation of ion conductivity; I did not consider all possible ion movements, rather only the important phenomena of adsorption and desorption. I did not concern ourselves with H_3_O^+^ status during adsorption and also assumed that desorption status was identical to that of the conductive state. In other words, I focused on direct measurement of H_3_O^+^ movements. I thus identified three cation states: adsorbed, conductive, and out-of-calculation. The potential difference between SO_3_^−^ and H_3_O^+^ drives the attraction that culminates in adsorption. When external forces (atomic or molecular interactions) are present, H_3_O^+^ may be in a conductive state.

**Figure 3:**
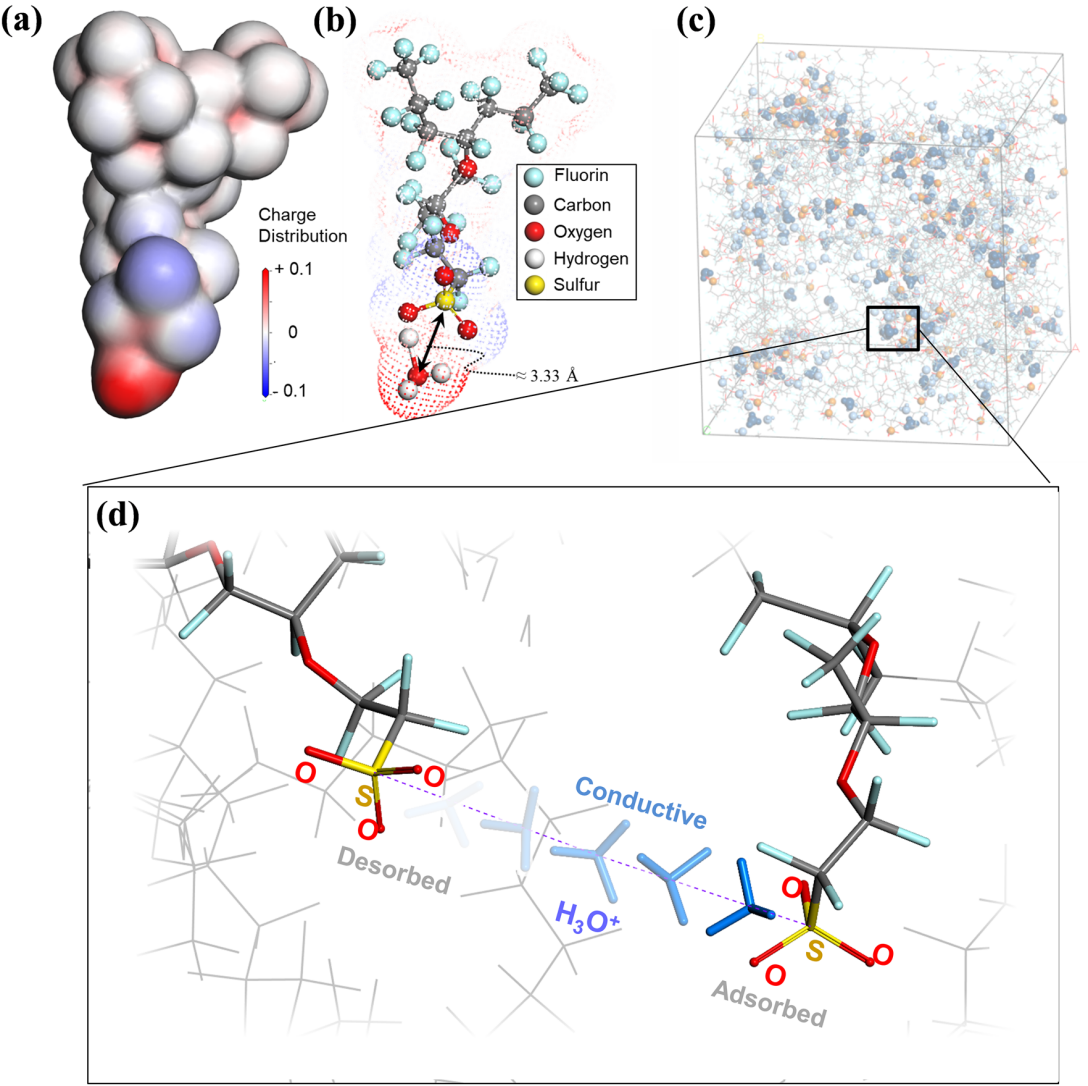
(**a**) A snapshot of charge distribution, and (**b**) structure of side chain with hydronium representing the adsorption distance; (**c**) the system of molecular dynamics simulation for the PEMFC. (**d**) The distance criterion used to evaluate the state of a cation (H_3_O^+^) near a negatively charged sulfur atom. Cation status (adsorbed or conductive) was determined by reference to the distance with the lowest energy level, as revealed by density functional theory. Cations associated with larger distances from the sulfur atom were considered to be ineffective and were excluded from calculation.

DFT calculation has been widely used to obtain the distance between two chemical or physical interacting atoms to be stable. Since adsorption was defined by the bonding distance in this study, DFT calculations were carried out to determine the adsorption distance based on the fundamental behavior of H_3_O^+^ around the negatively charged sulfonated groups. The DFT results reveal that the distances between sulfur and oxygen atoms with the lowest interaction energy are ranging from 3.32 to 3.33 Å according to the combinations of exchange–correlation functional (GGA-PBE and B3LYP) and polarization functions (DNP and TNP). Because of the natural atomic vibration in the adsorbed state, the threshold distance of the adsorption was set to be 3.5 Å which is slightly greater than that of the lowest energy state (3.32–3.33 Å). Figure 3a,b show the charge distribution and the optimized structure of side chain with hydronium, respectively.

The distribution of sulfur is one of the most critical factors to clarify the mechanism of ionic conduction affected by the morphology of side chain resulting from the migration of positively charged hydronium from or to negatively charged sulfonate groups. The distribution of sulfur atoms was calculated to find the distance between the sulfur clusters, and to determine the cut-off distance for exclusive hydronium considering the complex polymeric system with high density.

**Figure 4:**
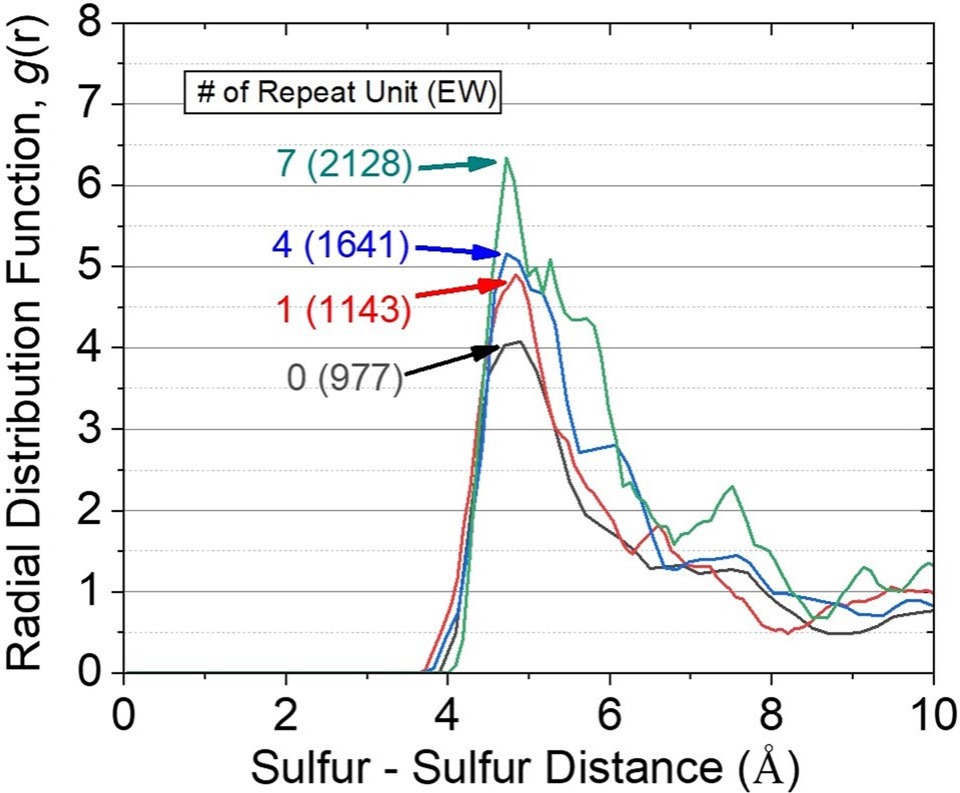
Radial distribution function (RDF) of sulfur–sulfur for the membrane with various length of side chains. The first peak for RDF of sulfur at the end of the longest side chain is 1.5 times higher than that of the shortest.

By dividing the count of the sulfur atoms by the volume of these spherical shell to achieve to a numerical density, it enables to obtain radial distribution function *g*(*r*). The mathematical formula is described as below:

4 $$ g(r) = \frac{n\left( r \right)}{{\rho {\kern 1pt} \cdot 4\pi {\kern 1pt} r^{2} \Delta r}} $$

where *n*(*r*) is the mean number of atoms in a shell of width *Δr* at distance *r*, *ρ* is the mean atom density. The sulfur atoms of the longer side chain were more distributed in sulfur cluster comparing to the shorter that the first peak for RDF of sulfur at the end of the longest side chain is 1.5 times higher than that of the shortest (Fig. 4). Based on the measure of H_3_O^+^ intra- or inter-polymer chain movements using a suggested new algorithm (Supplementary Figs. S4–S6), I possibly estimated that sulfur clusters for the long side chain contain more sulfur atoms from same chain. In addition, the short side-chains induced more inter-chain cation movement than longer side-chains. The proportion of cations moving between chains was 11.78% for the shortest side-chain, falling to 6.89, 6.51, and 5.15% when there were 1, 4, and 7 repeating units, respectively.

In this study, I postulated that the hydronium only moves to nearest-neighbor sulfonated group because the time scale of the simulation is too short to describe a moving cation from one sulfur cluster to the long-distance cluster over 8 Å. A H_3_O^+^ ion lying within the adsorption distance was regarded as adsorbed, and an ion up to 8 Å distant was considered conductive. However, any H_3_O^+^ ion lying more distant from a sulfur atom was ignored to minimize confusion, and only H_3_O^+^ ions < 8 Å distant from sulfurs were included in the calculations.

### Effect of side-chain length on ionic conductivity

**Figure 5:**
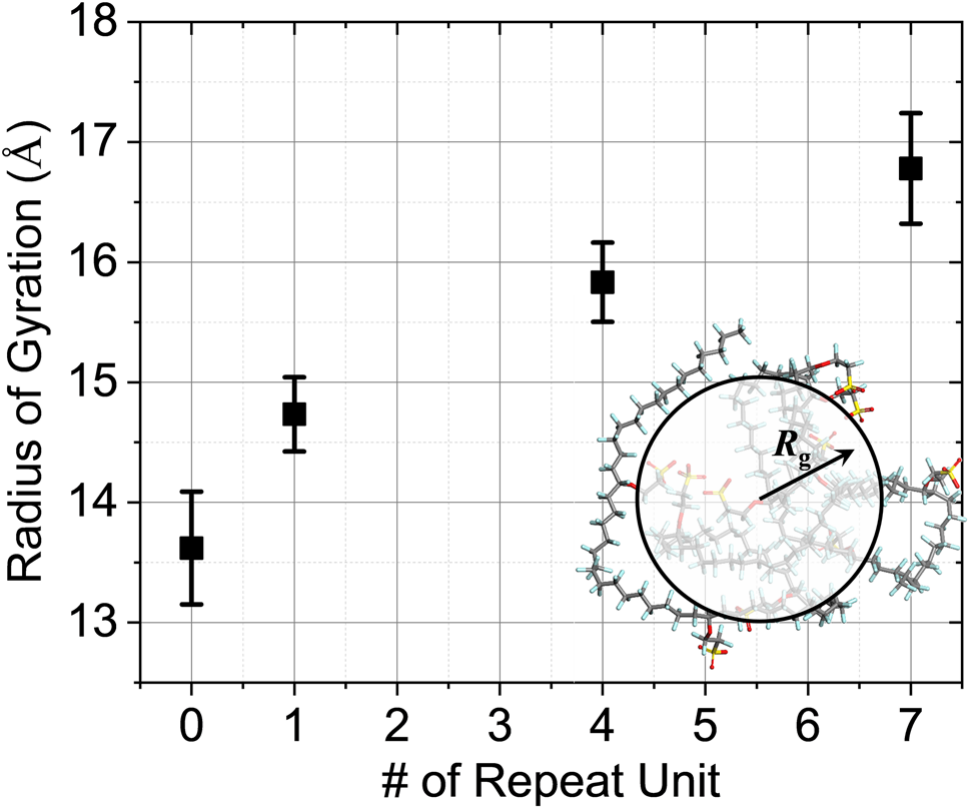
The radius of gyration increases with the molecular weight, represented by the length of side chain. The longer side chain with a stronger hydrophilic group has higher *R*_g_ than shorter side chains because of the hydrophobicity of the backbone.

In general, a single polymeric chain gyrates to ensure energy stabilization, and identical atoms, such as sulfurs at the ends of side-chains, associate according to their natural affinity. The radius of gyration (*R*_*g*_) is a basic concept for the features of polymer structures, which is defined as the root-mean-square (RMS) distance of the collection of atoms in the molecule from their common center of mass, and calculated from the following equation:

5 $$ R_{g}^{2} = \sum\limits_{i = 1}^{N} {m_{i} s_{i}^{2} } /\sum\limits_{i = 1}^{N} {m_{i} } $$

where *m*_*i*_, *s*_*i*_, and *N* are mass of atom *i*, the distance of atom *i* from the center of mass, and the total number of atoms, respectively. The radius of gyration increases with the molecular weight representing the length of side chain (Fig. 5). Since the difluoromethylene group tends to aggregate in water due to the hydrophobicity of the backbone, the longer side chain with a stronger hydrophilic group has higher *R*_*g*_ than shorter side chains.

Therefore, polymeric behavior impacts on ion conductivity. As the polymer backbone was –CF–, the side-chain length and backbone attachment position constituted the principal determinants of physical membrane properties. Many studies have explored the structural effects of side-chains on the diffusion that precedes ion conductivity^25–27,52^. While the result obtained from computational simulations showed the critical influence of the side chain on proton dissociation and transport under conditions of low hydration^18,19^, experimental results reveal that that short side-chains did not significantly affect water diffusion, proton transport, or hydrophobic-hydrophilic separation^27^. The results are confusing; it is premature to conclude that side-chain structure affects ion conductivity.

**Figure 6:**
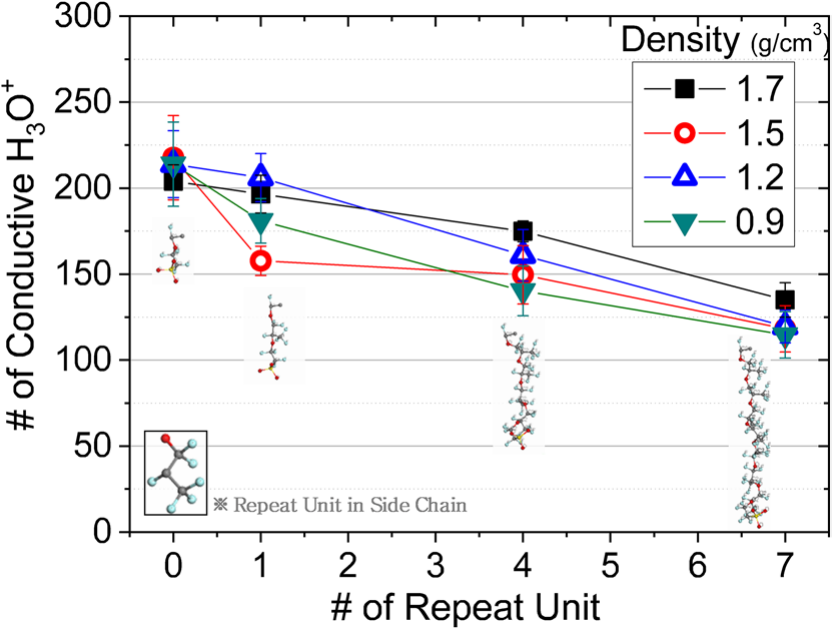
Structural variations in PFSA side-chains affect the conductive state of hydronium. Shorter side-chains were associated with more conductive H_3_O^+^. Irrespective of membrane density, the morphologies of the chain and side-chains significantly affected ionic movement.

I focused on the morphological features of the Nafion membrane that affect ion conductivity; however, these remain unclear and even basic details are lacking. I intuitively calculated ion conductivity by directly counting the numbers of H_3_O^+^ ions moving into the conductive state, as revealed by diffusion calculations. Figure 6 shows that the number of motile H_3_O^+^ ions decreased as side-chain length increased. The structural indices of the Nafion side-chain are *x* = 1 and *y* = 1; I varied the number of repeating units (i.e., index *y* of “Numerical evaluation of ion conductivity” section) in the side-chain from 0 to 1, 4, and 7 (Fig. 1b); *x* was held at 1. When the side-chains were shorter than those of Nafion, the number of motile (conducting) cations increased by up to 3.80%, but decreased by up to 31.32% when *y* was 0 or 7. Although the side-chain length is often used to quantify effects on ionic conductivity, it is difficult to hold the length constant, given the natural vibration associated with interactions between surrounding atoms.

I performed simulations at various system densities (0.9 to 1.7 g/cm^3^) to explore the effect of free space on cation motion. The various densities for the simulation system reflects a free space change; more free space should enhance more ions in conductive state. I found no marked difference among systems of varying density since there were only the volumetric (or free space) differences surrounding cluster formation of the polymer resulting from the ionic clustering, not the deformation of polymer chains with hydronium and water.

Thus, only the density of the entire system, rather than any direct effect of density on single chains, affected ionic conductance. Any direct effect of density (and thus free volume) on H_3_O^+^ conductivity will be evaluable only when the influence of free space on structural changes within a single polymeric chain is known. Practically, it is hard to investigate all the possible cases of combined polymeric morphology composing the system, so which was carried out more than 10 cases for the each of the simulation system to improve the reliability of the results.

### Mechanism of ion conduction

I found that shorter side-chains enhanced cation movement, as described in “Effect of side-chain length on ionic conductivity” section. However, more data are required; it is necessary to trace particle movements to clarify how cations travel within PEMFCs with complicated structures. To optimize ion conductivity, cations require channels that link chains to other chains, not channels within chains, while cations in polymer membranes with highly hydrated condition move through H_2_O clusters that form transport channels^15,41,42^.

**Figure 7:**
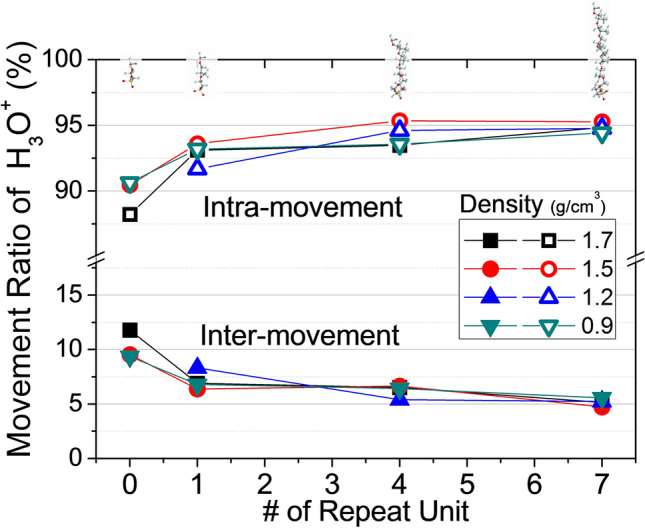
Structural variations affect the intra-/inter-movement ratio of ions in a PFSA membrane. H_3_O^+^ intra-movement increases with increasing side-chain length, because this fundamentally affects both the surface area and free volume associated with chain gyration.

I measured H_3_O^+^ intra- or inter-polymer chain movements using a simple new algorithm (Supplementary Figs. S4–S6). I explored whether ion conductivity was principally inter- or intra-chain; this aids the design of membranes with optimal ion conductivity. Figure 7 shows that side-chain length affects ion conductivity; short side-chains induced more inter-chain cation movement than longer side-chains. The proportion of cations moving between chains was 11.78% for the shortest side-chain, falling to 6.89, 6.51, and 5.15% when there were 1, 4, and 7 repeating units, respectively. The polymer used in the simulation had 10 uniformly spaced side-chains per polymer chain, and tended to gyrate to stabilize the energy state. However, polymer chains with short side-chains failed to cluster sulfur atoms via gyration because the chain length was inadequate. Thus, most of the cation movement was inter- rather than intra-chain.

For membranes with polymer chains featuring long side-chains, intra-polymer movements constituted 95% of all motions, and inter-polymer movements 5%. In particular, a polymer chain with seven repeating side-chain units was associated with 5% more intra-chain cation movements than a chain with the shortest unit. When simulating ion conductivity, I assumed that the bonding distance between each side-chain was uniform, and that all polymer chains were identical. Although these assumptions limit the direct use of our data in PEMFC design, I believe that ion conductivity within a PEM requires side-chains containing negatively charged sulfur atoms. Intra-chain movement attained 95% within membranes with long side-chains; inter-chain movement was low. Inter-chain movement inside the membrane with the shortest side-chain was 6.63% greater than that within the membrane with the longest side-chain.

## Conclusions

Rapid expansion of the fuel cell market renders it essential to understand membrane fundamentals. Here, I performed MD simulations of PFSA-based membranes to explore morphological effects on ion conductivity estimated using an intuitive, quantitative method. I focused on whether hydroniums were adsorbed to or desorbed from negatively charged sulfonate groups; I employed conventional calculations featuring MSDs that included natural atomic vibrations. Shorter side-chains allowed H_3_O^+^ ions to persist for longer in the conductive state, enhancing ion conductivity. In addition, the hydronium proton tracking showed that shorter side-chains allowed hydroniums to move among host groups, as suggested in earlier studies. Therefore, shorter side-chains enhance ion conductivity.

## Supplementary information

Supplementary Information.
