# Supplementary material for: Morphological effect of side chain on H3O+ transfer inside polymer electrolyte membranes across polymeric chain via molecular dynamics simulation
Source: Sci Rep. 2020 Dec 16;10:22014. doi: 10.1038/s41598-020-77971-6 (PMC7745029; doi:10.1038/s41598-020-77971-6)
Supplement: Supplementary file 1 — Supplementary Information. [file 41598_2020_77971_MOESM1_ESM.doc]

**Supplementary information for “Morphological effect of side chain on H_3_O^+^ transfer inside polymer electrolyte membranes across polymeric chain via molecular dynamics simulation”**

**JinHyeok Cha**

Institute of Fundamentals and Advanced Technology, Hyundai Motor Company, 37 Cheoldobangmulgwan-ro, Uiwang-si, Gyeonggi-do 16082, Republic of Korea

**Abstract**

I provide here supplementary data on the details of the force field and parameters, charge distributions of Nafion, H_3_O^+^, and H_2_O, and the concept of intra-/inter-movement based on the molecular behaviors of various states.

**Details of the force field**

Ie employed the DREIDING potentials for molecular dynamic simulations; these were developed by the Goddard group^1,2^ and the Daggett group^3^ and include the following bond-stretching (*E_b_*), angle-bending (*E_a_*), torsion (*E_t_*), and van der Waals (*E*_VDW_) terms:

$$E_{b}(R)=\frac{1}{2}K_{b}(R-R_{0})^{2}$$

$$E_{a}(\theta)=\frac{1}{2}K_{\theta}(\theta-\theta_{0})^{2}$$

$$E_{t}(\varphi)=\sum_{n} \frac{1}{2}V_{n}\left[1-d_{n}cos(n\varphi)\right]$$

$$E_{VDW}(R)=D_{0}\left[\left(\frac{R_{1}}{R}\right)^{12}-2\left(\frac{R_{1}}{R}\right)^{6}\right]$$

All interaction parameters used to calculate DREIDING potentials are shown in Tables S1 and S2.

**References**

1. Mayo, S. L.; Olafson, B. D.; Goddard, W. A. DREIDING: A Generic Force Field for Molecular Simulations. *J. Phys. Chem.* **94** (26), 8897–8909 (1990).

2. Jang, S. S.; Molinero, V.; Çaǧin, T.; Goddard, W. A. Nanophase-Segregation and Transport in Nafion 117 from Molecular Dynamics Simulations: Effect of Monomeric Sequence. *J. Phys. Chem. B* **108**, 3149–3157 (2004).

3. Levitt, M.; Hirshberg, M.; Sharon, R.; Laidig, K. E.; Daggett, V. Calibration and Testing of a Water Model for Simulation of the Molecular Dynamics of Proteins and Nucleic Acids in Solution. *J. Phys. Chem. B* **25**, 5051 (1997).

Table S1. Parameters used to evaluate bond interactions.

| Bond | *K_b_* | *R*_0_ | Angle | *K_θ_* | *θ*_0_ | Torsion | Parameter |
| --- | --- | --- | --- | --- | --- | --- | --- |
| S−O | 700.0 | 1.48 | O−S−O | 350.0 | 115.50 | X−C−C−X | V_3_(d_3_): 2.0000 (-1) |
| S−C^2^ | 700.0 | 1.80 | O−S−C^2^ | 350.0 | 109.47 | C^1^−C^1^−C^1^−C^1^ | V_3_(d_3_): 6.4342 (-1) |
| C^1^−C^1^ | 429.320 | 1.4982 | S−C^2^−F | 100.0 | 109.47 | F−C^1^−C^1^−C^1^ | V_3_(d_3_): 8.2444 (1) |
| C^1^−C^2^ | 700.0 | 1.53 | X−C−X | 100.0 | 109.47 | F−C^1^−C^1^−F | V_3_(d_3_): 8.0848 (-1) |
| C^2^−C^2^ | 700.0 | 1.53 | C−C−C | 106.27 | 122.55 | X−C−O−X | V_3_(d_3_): 2.0000 (-1) |
| C−F | 605.259 | 1.336 | C−C−F | 100.34 | 118.32 | X−C−S−X | V_3_(d_3_): 2.0000 (-1) |
| C^2^−O | 700.0 | 1.42 | F−C−F | 108.24 | 121.50 |  |  |
| H^h^−O^h^ | 1085.96 | 0.982 | H^h^−O^h^−H^h^ | 79.03 | 113.40 |  |  |
| H^w^−O^w^ | 500.0 | 1.00 | H^w^−O^w^−H^w^ | 120.0 | 109.47 |  |  |

The units of *K_b_*, *R*_0_, *K_θ_*, *θ*_0_, and *V*_n_ are kcal/mol/Å, Å, kcal/mol/rad^2^, degrees, and kcal/mol, respectively.

Table S2. Parameters used to calculate van der Waals interactions.

| Atom type | *D*_0_ (kcal/mol) | *R*_0_ (Å) |
| --- | --- | --- |
| S | 0.344 | 4.03 |
| O (membrane) | 0.0957 | 3.4046 |
| C^1^ | 0.0844 | 3.8837 |
| C^2^ | 0.0951 | 3.8983 |
| F | 0.0496 | 3.3953 |
| O^h^, O^w^ | 0.1848 | 3.5532 |
| H^h^, H^w^ | 0.01 | 0.90 |


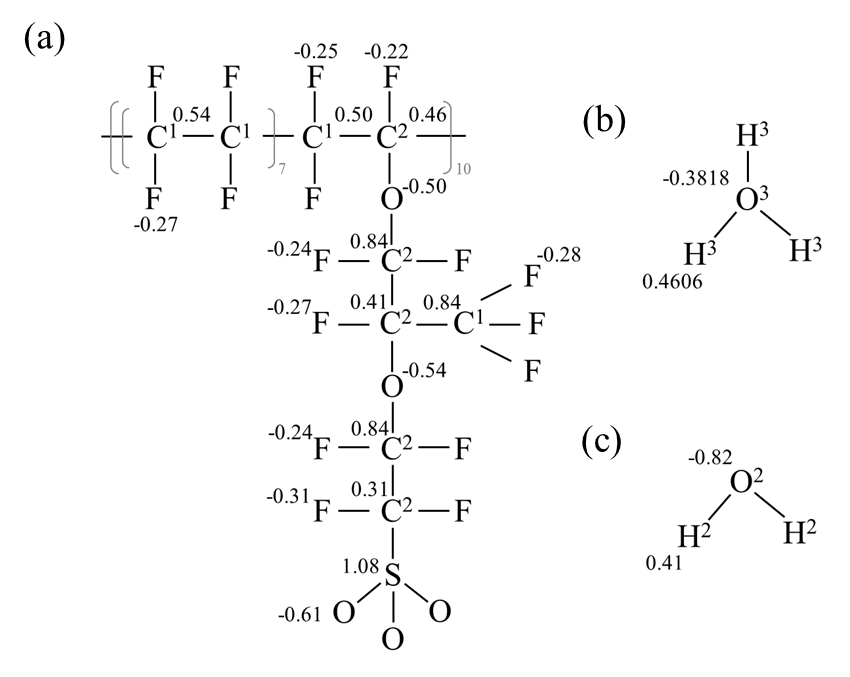


Figure S1. Charge distributions for (a) a monomer with an index (*x* = 1, *y* = 1) corresponding to Nafion; (b) H_3_O^+^; and, (c) H_2_O.


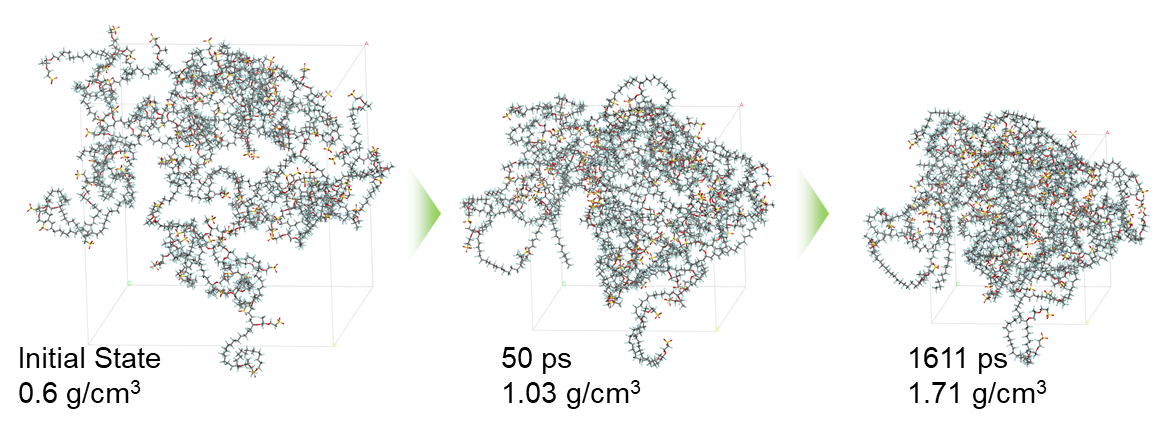


Figure S2. Snapshots of annealing process to build PEMFC system with the density from 0.6 g/cm^3^ of initial state up to 1.7 g/cm^3^ by *NPT* ensemble to minimize energy of the system effectively.


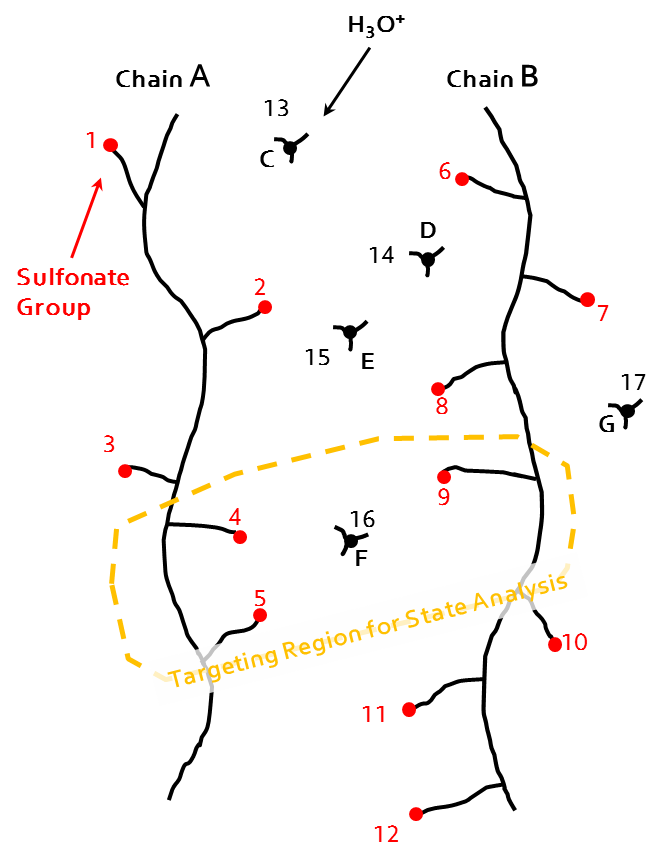


Figure S3. Molecular behavior inside a membrane was used to define the state of adsorption in terms of inter-movement and intra-movement. The selected region (yellow dashed line) is used to describe these movements in Figures S3 and S4.


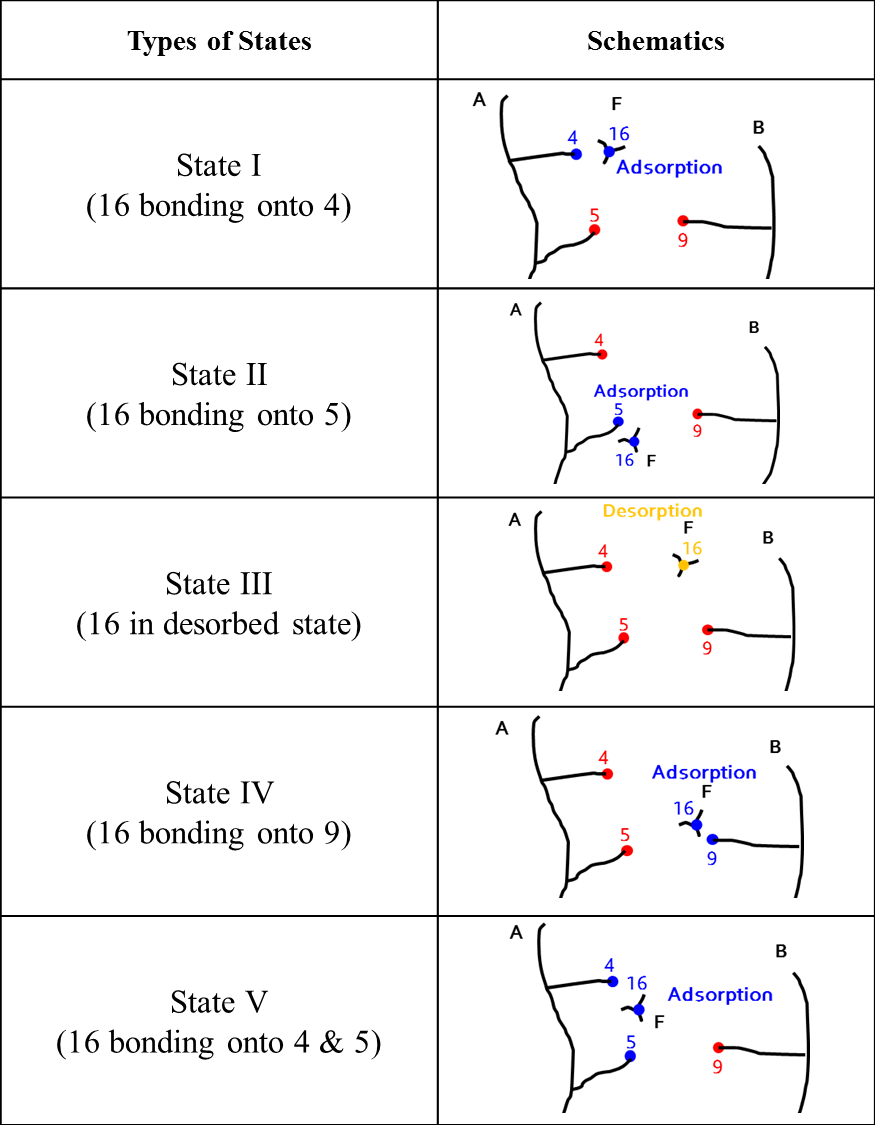


Figure S4. Five possible cases of molecular adsorption (from the selected region of Fig. S3). We divided the states into five different cases and considered whether molecule 16 was adsorbed to sulfur 4, 5, or 9, or was desorbed.


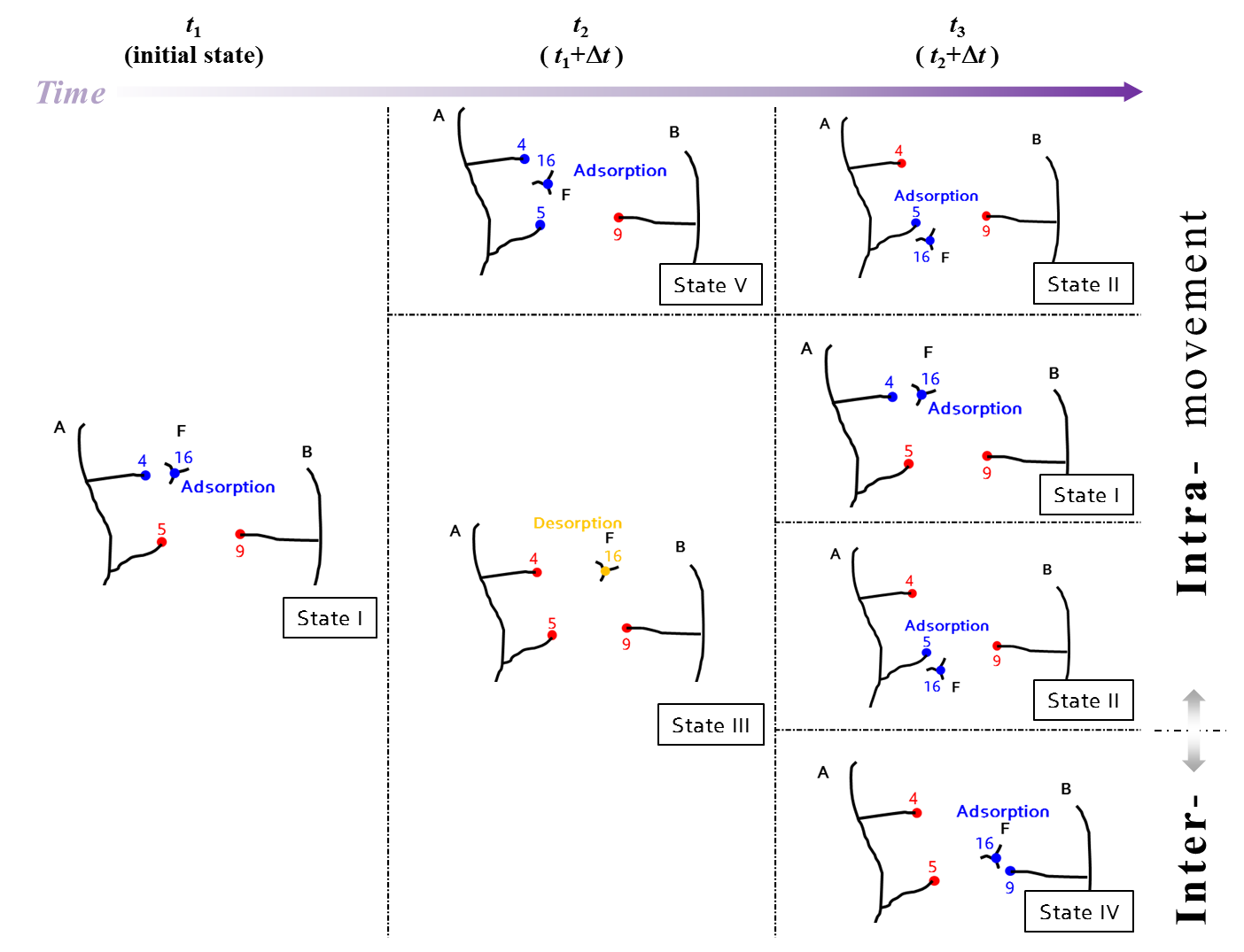


Figure S5. The concept used to describe the molecular behaviors of various states. The simulation time steps run from *t*_1_ to *t*_2_, and then to *t*_3_; the hydronium ion moves from the sulfur molecule of one chain to that of others. Initially, state I potentially becomes state III or V; adsorption of molecule 16 of state V to sulfur 5 (corresponding to state II) is an intra-movement. On the other hand, when molecule 16 of state III becomes adsorbed to sulfur 4 or 5 (corresponding to state I or II, respectively), intra-movement is occurring. In addition, molecule 16 in state III moving to sulfur 9 in state IV is an example of inter-movement.
